# Increased Incidence of Invasive Haemophilus influenzae Disease Driven by Non-Type B Isolates in Ontario, Canada, 2014 to 2018

**DOI:** 10.1128/Spectrum.00803-21

**Published:** 2021-10-06

**Authors:** Lisa R. McTaggart, Kirby Cronin, Chi Yon Seo, Sarah Wilson, Samir N. Patel, Julianne V. Kus

**Affiliations:** a Public Health Ontario, Toronto, Ontario, Canada; b National Microbiology Laboratory, Public Health Agency of Canada, Winnipeg, Manitoba, Canada; c Dalla Lana School of Public Health, University of Toronto, Toronto, Ontario, Canada; d ICES, Toronto, Ontario, Canada; e Department of Laboratory Medicine and Pathobiology, University of Toronto, Toronto, Ontario, Canada; Griffith University

**Keywords:** *Haemophilus influenzae*, serotype, invasive disease, antimicrobial susceptibility

## Abstract

Haemophilus influenzae can cause serious invasive disease. We report the epidemiology and antimicrobial susceptibility of invasive H. influenzae in Ontario, Canada, from 2014 to 2018 from laboratory-based data. Blood was the most common specimen source (89.5%). Consistent with widespread vaccination against serotype b (Hib), the incidence of Hib in Ontario remained low (0.04 cases per 100,000 population). H. influenzae disease primarily afflicted those <1 and ≥65 years of age. From 2014 to 2018, cases of invasive H. influenzae increased 5.6%, from 1.67 to 2.06 cases per 100,000 population, the majority of which were attributed to a 7.6% increase in the incidence of H. influenzae in those ≥65 years old. H. influenzae disease was primarily caused by nontypeable H. influenzae (NTHi) (74.2%) and, to a much lesser extent, serotype a (Hia) (8.9%) and serotype f (Hif) (10.2%). Serotype-dependent trends in antimicrobial susceptibility were observed. Hia and Hif isolates were predominantly susceptible to all antibiotics tested, while 27.2% of NTHi isolates were nonsusceptible to ampicillin. Resistance to ceftriaxone and meropenem, first-line antibiotics for invasive disease treatment, was nonexistent. The incidence of invasive H. influenzae in Ontario is increasing. The incidence and antimicrobial susceptibility of all serotypes and nontypeable H. influenzae should be monitored.

**IMPORTANCE** H. influenzae can cause serious invasive, life-threatening disease and is considered 1 of 12 priority pathogens by the World Health Organization. Widespread vaccination against H. influenzae serotype b (Hib) has resulted in very low incidence of Hib in Ontario and other regions that have vaccination programs. However, the epidemiology of non-Hib serotypes and nontypeable H. influenzae (NTHi) remains poorly understood. Here, we describe the epidemiology of all invasive H. influenzae isolates (N = 1,338) received by our laboratory over the 5-year period and report on the antimicrobial susceptibility patterns by serotype. Overall, we observed an increase in the incidence of invasive disease over the study period, primarily driven by NTHi. Serotype-dependent trends in antimicrobial susceptibility were also observed. This work contributes to the global understanding of H. influenzae epidemiology and antimicrobial resistance and is additionally important for further vaccine planning initiatives.

## INTRODUCTION

Haemophilus influenzae is a nonmotile, Gram-negative, facultatively anaerobic coccobacillus capable of causing infections in humans ranging from noninvasive acute otitis media to severe invasive infections such as meningitis, epiglottis, pneumonia, and septicemia (1, 2). H. influenzae strains are characterized as encapsulated, which can be serotyped as a (Hia), b (Hib), c (Hic), d (Hid), e (Hie), f (Hif), or unencapsulated nontypeable (NTHi). Although Hib was historically a common cause of severe invasive infection in children, the introduction of Hib vaccines in the 1980s resulted in a precipitous drop in Hib disease in countries with Hib immunization programs (3).

Concomitant with the decline in invasive Hib disease, several studies have documented the emergence of invasive H. influenzae infections caused by non-Hib strains, particularly NTHi (4–6). Rather than occurring primarily as a childhood disease, the epidemiology of invasive H. influenzae infections disproportionately affects older adults (4–11). Finally, the incidence of antibiotic resistance among H. influenzae is increasing, particularly to ampicillin. Isolated reports of resistance to cephalosporins and carbapenems warrant vigilant surveillance of antimicrobial susceptibility (12–14).

Prior to mid-2018, the only provincially reportable invasive H. influenzae infections in Ontario were those caused by Hib. As of 1 May 2018, invasive disease caused by all serotypes of H. influenzae as well as NTHi became provincially reportable as diseases of public health significance (15). The purpose of this study is to detail the epidemiology and antimicrobial susceptibility of H. influenzae from invasive cases in Ontario received at the Public Health Ontario Laboratory (PHOL) from 2014 to 2018. With ∼40% of Canada’s population, Ontario is the country’s most populous province (16), with a vast geography that includes both densely populated urban areas and remote and isolated geographies in northern Ontario. Understanding the current epidemiology and antimicrobial susceptibility of H. influenzae is crucial for aiding physicians in the diagnosis and treatment of these serious invasive infections and informing future vaccine development.

## RESULTS

A total of 1,417 isolates of H. influenzae were received by PHOL from 2014 to 2018. Of these, 79 were excluded because they were derived from nonsterile sites, leaving 1,338 invasive isolates of H. influenzae. To capture single infection episodes only, 57 isolates were excluded because they represented additional subsequent isolates of the same serotype from the same individual received within 6 months of the initial isolate. The final data set contained 1,281 cases of invasive H. influenzae in Ontario, each assigned to a year from 2014 to 2018 based on the date the first isolate was received by PHOL. Of these, 1,273 were serotyped. Eight were not serotyped and were excluded from all serotyping analyses but retained in all other analyses.

### Specimen type and serotype.

Of the isolates, 89.5% (1,197/1,338) were from blood and 3.3% (44/1,338) were from cerebrospinal fluid (CSF). Other sources included biopsy specimens, pleural fluid, lung tissue, joint fluid, and peritoneal fluid. A significant association was noted between the serotype and specimen type (blood, CSF, and other) (χ^2^ = 18.3, *P* = 0.019). A significantly greater proportion of NTHi strains were isolated from specimens other than blood and CSF (7.9%) than were other serotypes (*P* = 0.04), and more Hif strains were isolated from blood (98.5%) than were other serotypes (*P* = 0.009) (Table 1). There was also a significant association between the specimen type and age (χ^2^ = 90.1, *P* < 0.001); although blood was a predominant specimen type in infants <1 (86.1%), CSF specimens were disproportionately higher in this age group (10.1%, *P* = 0.009) compared to other age groups (1 to 19, 20 to 39, 40 to 64, and ≥65 years of age).

**TABLE 1:** Distribution of specimen types from which invasive H. influenzae were isolated by serotype^*a*^

Serotype^*b*^	*n*	Data for specimen type (*n* [%])		
		Blood	CSF	Other
NTHi	986	874 (88.6)	34 (3.4)	**78 (7.9)**
Hia	127	112 (88.2)	6 (4.7)	9 (7.1)
Hib	33	31 (93.9)	1 (3.0)	1 (3.0)
Hie	50	48 (96.0)	2 (4.0)	0 (0)
Hif	133	**131 (98.5)**	1 (0.8)	1 (0.8)

a The data represent all isolates, including multiple isolates per infection episode, for which serotype data were available (*n* = 1,329). Statistically significant (*P* ≤ 0.05) trends are indicated in bold.

b The single Hid isolate was omitted from this analysis.

**FIG 1:**
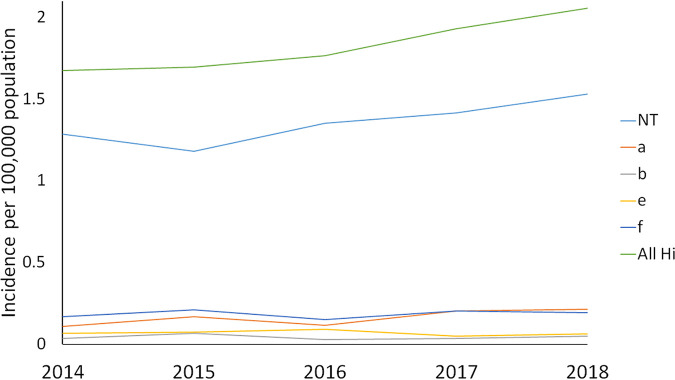
Incidence of invasive H. influenzae disease, showing single infection episodes by serotype in Ontario, 2014 to 2018.

From 2014 to 2018, the incidence of all invasive H. influenzae disease in Ontario, based on 1,281 single infection episodes, increased from 1.67 to 2.06 cases per 100,000 population, with a significant annual percent change (APC) of 5.6% (*P* = 0.006). The majority of cases were nontypeable (NTHi) (*n* = 950, 74.2%), with 114 cases of Hia (8.9%), 30 cases of Hib (2.3%), 1 case of Hid (0.1%), 48 cases of Hie (3.8%), and 130 cases of Hif (10.2%). Mirroring the temporal trend in incidence of H. influenzae, the incidence of NTHi increased from 1.29 to 1.53 cases per 100,000 population from 2014 to 2018; however, the APC of 5.5% was not significant (*P* = 0.054). Incidences of serotypes a through f remained stable (*P* > 0.05), and the overall incidence of Hib from 2014 to 2018 was low, at 0.04 cases per 100,000 population (Fig. 1).

### Age.

**FIG 2:**
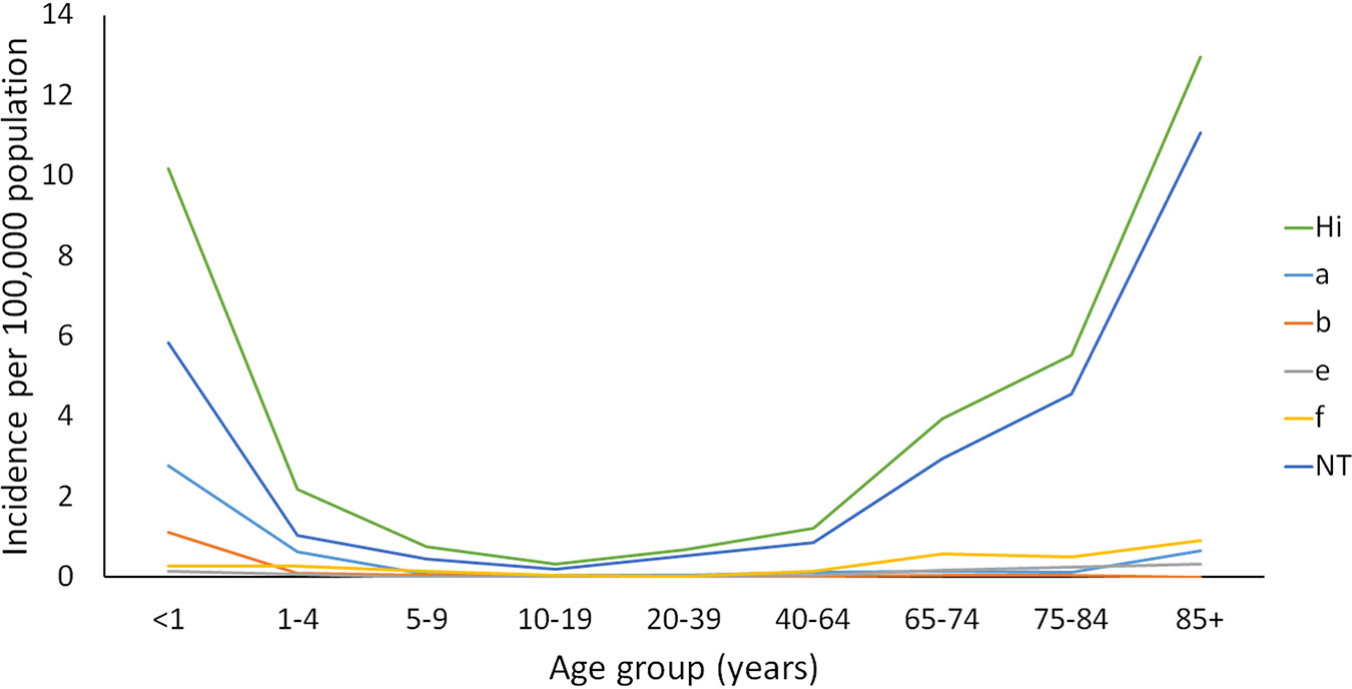
Incidence of invasive H. influenzae disease, showing single infection episodes by serotype and age group in Ontario, 2014 to 2018.

While the age of individuals with invasive H. influenzae infection ranged from less than 1 to 102 years of age, children <1 and individuals ≥85 years of age had the highest incidence rates of H. influenzae, at 10.17 and 12.97 cases per 100,000 population, respectively. The elderly age groups 65 to 74 and 75 to 84 years of age also had higher incidence rates of H. influenzae, at 3.94 and 5.54 cases per 100,000 population, respectively (Fig. 2).

Among the ≥65 age group, the majority of invasive infections were caused by NTHi (80.7%) and Hif (10.8%) (Table 2). Of the invasive H. influenzae infections in children <1 year of age, 57.5% were attributed to NTHi (5.85 cases per 100,000 population), 27.4% to Hia (2.79 cases per 100,000 population), and 11.0% to Hib (1.11 cases per 100,000 population) (Table 2, Fig. 2). There was a statistically significant association between age group and H. influenzae serotype distribution (χ^2^ = 135.8, *P* ≤ 0.001), with a statistically greater proportion of NTHi (*P* < 0.001) and fewer Hia (*P* < 0.001) cases derived from ≥65 year olds than other age groups. Conversely, Hia disease was statistically proportionally higher among the <1 (*P* < 0.001) and 1 to 19 (*P* < 0.001) age groups compared to other age groups, while NTHi infection was disproportionately lower (<1 age group, *P* = 0.017; 1 to 19 age group, *P* < 0.001) (Table 2).

**TABLE 2:** Distribution of serotypes of H. influenzae among age groups, 2014 to 2018^*a*^

Age	*n*	Data for serotype (*n* [%])					
		NTHi	Hia	Hib	Hid	Hie	Hif
<1	73	**42 (57.5)**	**20 (27.4)**	**8 (11.0)**	0 (0.0)	1 (1.4)	2 (2.7)
1–19	115	**63 (54.8)**	**25 (21.7)**	7 (6.1)	0 (0.0)	3 (2.6)	17 (14.8)
20–39	130	104 (80.0)	11 (8.5)	4 (3.1)	0 (0.0)	5 (3.8)	6 (4.6)
40–64	296	210 (70.9)	33 (11.1)	6 (2.0)	1 (0.3)	12 (4.1)	34 (11.5)
≥65	646	**521 (80.7)**	**24 (3.7)**	5 (0.8)	0 (0.0)	26 (4.0)	70 (10.8)

a The data represent single infection episodes of H. influenzae for which age and serotype data were available (*n* = 1,260). Statistically significant (*P* ≤ 0.05) trends are indicated in bold.

We examined the data for any temporal changes in age-associated infections with H. influenzae and all serotypes. While no age group <65 years of age (<1, 1 to 19, 20 to 39, or 40 to 64 years of age) showed a change in the incidence of invasive H. influenzae infections between 2014 and 2018, the incidence of H. influenzae in persons ≥65 years of age increased significantly from 4.95 to 6.46 cases per 100,000 population with an APC of 7.6% (*P* = 0.004). The increase in H. influenzae incidence in this population was attributed to nonsignificant increases in Hif and NTHi infection and a significant increase in Hia infection (APC 24.3%, *P* = 0.046). Although the overall incidence of invasive H. influenzae infections in those 20 to 39 years of age remained unchanged from 2014 to 2018, the incidence of Hia disease increased significantly, with an APC of 43.3% (*P* = 0.004), from 0.03 to 0.10 cases per 100,000 population. There were no statistically significant temporal changes in serotype incidence for any other age group (<1, 1 to 19, or 40 to 64 years of age).

### Geography.

Geographic analysis was performed on 905 of the 1,281 cases of H. influenzae (70.6%) where the postal code of the patient’s residence was available and used to assign the case to one of Ontario’s 34 public health units (PHUs). Mapping of the incidence of H. influenzae by PHU showed little variation in the geographic distribution; cases were reported in all regions of the province, although higher incidence rates were noted in the PHUs surrounding Georgian Bay and in southwestern Ontario (Fig. S1A in the supplemental material). We also specifically examined the geographic distribution of cases of NTHi in individuals aged ≥65 years; such cases were reported in all regions of the province (Fig. S1B).

### Antimicrobial susceptibility testing.

Data on antimicrobial susceptibility testing (AST) and beta-lactamase were available for 99.1% (1,261/1,273) and 99.2% (1,263/1,273) of serotyped isolates, respectively. Serotype-associated patterns were apparent in the AST and beta-lactamase data (Table 3). The majority of Hia and Hif isolates were susceptible to most antimicrobials tested. In contrast, a statistically significantly greater proportion of Hib (20.0%), Hie (16.7%), and NTHi (27.2%) isolates were nonsusceptible to ampicillin (*P* ≤ 0.05) (Table 3). Rates of beta-lactamase activity (Hia, 2.6%; Hib, 20.0%; Hie, 16.7%; Hif, 0.8%; and NTHi, 26.9%) mirrored those of ampicillin nonsusceptibility, with 99.3% (271/273) of ampicillin-nonsusceptible isolates overall demonstrating beta-lactamase activity. A statistically greater proportion of Hie (54.2%) and Hib (16.7%) isolates were nonsusceptible to clarithromycin and tetracycline, respectively, than the other typeable and nontypeable H. influenzae isolates (*P* < 0.01) (Table 3). As well, a greater proportion of Hib (43.3%), Hie (22.9%), and NTHi (27.4%) isolates were nonsusceptible to trimethoprim-sulfamethoxazole compared to Hia (4.4%) and Hif (12.5%) isolates (*P* < 0.01). The single Hid isolate was susceptible to all antimicrobials except trimethoprim-sulfamethoxazole (MIC, >2/38). Overall, the percentage of isolates susceptible to meropenem (100%) and the third- and fourth-generation cephalosporins ceftriaxone (100%), cefixime (99.9%), and cefepime (99.8%) remained extremely high (Table 3).

**TABLE 3:** Antimicrobial susceptibility patterns of invasive H. influenzae in Ontario, 2014 to 2018

Antibiotic	Range (μg/ml)	MIC_50_ (μg/ml)	MIC_90_ (μg/ml)	% NS^*a*^
Serotype a (*n* = 114)				
Amoxicillin-clavulanate	≤2/1 to 4/2	≤2/1	≤2/1	0.0
Ampicillin	≤0.12 to >4	≤0.12	0.25	2.6
Ampicillin-sulbactam	≤1/0.5 to 2/1	≤1/0.5	≤1/0.5	0.0
Cefaclor	≤4 to >16	≤4	4	2.6
Cefepime	≤0.12 to 1	≤0.12	0.12	0.0
Cefixime	≤0.12	≤0.12	0.12	0.0
Ceftriaxone	≤0.03 to 0.06	≤0.03	0.03	0.0
Cefuroxime	≤0.5 to 4	≤0.5	1	0.0
Chloramphenicol	≤0.5 to 2	≤0.5	1	0.0
Clarithromycin	≤0.12 to 8	8	8	0.0
Imipenem	≤0.5 to 2	≤0.5	1	0.0
Levofloxacin	≤0.03 to 0.5	≤0.03	0.03	0.0
Meropenem	≤0.06 to 0.25	≤0.06	0.06	0.0
Sparfloxacin	≤0.03 to 0.5	≤0.03	0.03	0.9
Tetracycline	0.5 to 4	1	1	0.9
Trimethoprim-sulfamethoxazole	≤0.06/1.19 to >2/38	0.25/4.75	0.5/9.5	4.4
Serotype b (*n* = 30)				
Amoxicillin-clavulanate	≤2/1 to 4/2	≤2/1	≤2/1	0.0
Ampicillin	≤0.12 to >4	0.25	>4	20.0
Ampicillin-sulbactam	≤1/0.5 to 2/1	≤1/0.5	≤1/0.5	0.0
Cefaclor	≤4 to 8	≤4	4.4	0.0
Cefepime	≤0.12 to 1	≤0.12	0.12	0.0
Cefixime	≤0.12 to 0.25	≤0.12	0.12	0.0
Ceftriaxone	≤0.03	≤0.03	0.03	0.0
Cefuroxime	≤0.5 to 2	≤0.5	1	0.0
Chloramphenicol	≤0.5 to 1	1	1	0.0
Clarithromycin	1 to 8	4	8	0.0
Imipenem	≤0.5 to 1	≤0.5	≤0.5	0.0
Levofloxacin	≤0.03	≤0.03	≤0.03	0.0
Meropenem	≤0.06 to 0.12	≤0.06	≤0.06	0.0
Sparfloxacin	≤0.03 to 0.06	≤0.03	≤0.03	0.0
Tetracycline	0.5 to >4	0.5	>4	16.7
Trimethoprim-sulfamethoxazole	0.5/9.5 to >2/38	0.5/9.5	>2/38	43.3
Serotype e (*n* = 48)				
Amoxicillin-clavulanate	≤2/1	≤2/1	≤2/1	0.0
Ampicillin	≤0.12 to >4	0.25	>4	16.7
Ampicillin-sulbactam	≤1/0.5 to >2/1	≤1/0.5	≤1/0.5	2.1
Cefaclor	≤4 to >16	≤4	8	4.2
Cefepime	≤0.12 to 1	≤0.12	≤0.12	0.0
Cefixime	≤0.12 to 0.5	≤0.12	≤0.12	0.0
Ceftriaxone	≤0.03 to 0.12	≤0.03	≤0.03	0.0
Cefuroxime	≤0.5 to 4	1	1	0.0
Chloramphenicol	≤0.5 to 1	≤0.5	≤0.5	0.0
Clarithromycin	4 to 16	16	16	54.2
Imipenem	≤0.5 to 2	≤0.5	≤0.5	0.0
Levofloxacin	≤0.03 to 0.12	≤0.03	≤0.03	0.0
Meropenem	≤0.06 to 0.25	≤0.06	≤0.06	0.0
Sparfloxacin	≤0.03 to 0.06	≤0.03	≤0.03	0.0
Tetracycline	0.25 to 1	0.5	1	0.0
Trimethoprim-sulfamethoxazole	≤0.06/1.19 to >2/38	0.5/9.5	>2/38	22.9
Serotype f (*n* = 128)				
Amoxicillin-clavulanate	≤2/1 to 4/2	≤2/1	≤2/1	0.0
Ampicillin	≤0.12 to >4	≤0.12	0.25	0.8
Ampicillin-sulbactam	≤1/0.5 to >2/1	≤1/0.5	≤1/0.5	0.8
Cefaclor	≤4 to 8	≤4	≤4	0.0
Cefepime	≤0.12 to >2	≤0.12	0.25	1.6
Cefixime	≤0.12 to >1	≤0.12	≤0.12	0.8
Ceftriaxone	≤0.03 to 0.5	≤0.03	0.12	0.0
Cefuroxime	≤0.5 to 4	≤0.5	1	0.0
Chloramphenicol	≤0.5 to 1	≤0.5	≤0.5	0.0
Clarithromycin	0.12 to 16	8	8	6.3
Imipenem	≤0.5 to 1	≤0.5	≤0.5	0.0
Levofloxacin	≤0.03 to 0.12	≤0.03	≤0.03	0.0
Meropenem	≤0.06 to 0.25	≤0.06	≤0.06	0.0
Sparfloxacin	≤0.03 to 0.25	≤0.03	≤0.03	0.0
Tetracycline	0.25 to 1	0.5	0.5	0.0
Trimethoprim-sulfamethoxazole	≤0.06/1.19 to >2/38	0.25/4.75	1/19	12.5
Serologically nontypeable (*n* = 940)				
Amoxicillin-clavulanate	≤2/1 to 4/2	≤2/1	≤2/1	0.0
Ampicillin	≤0.12 to >4	0.25	>4	27.2
Ampicillin-sulbactam	≤1/0.5 to >2/1	≤1/0.5	2/1	4.3
Cefaclor	≤4 to >16	≤4	8	4.9
Cefepime	≤0.12 to >2	≤0.12	≤0.12	0.1
Cefixime	≤0.12 to 1	≤0.12	≤0.12	0.0
Ceftriaxone	≤0.03 to 0.5	≤0.03	≤0.03	0.0
Cefuroxime	≤0.5 to 8	≤0.5	1	0.2
Chloramphenicol	≤0.5 to 4	≤0.5	0.6	0.2
Clarithromycin	≤0.12 to >16	4	8	9.7
Imipenem	≤0.5 to >4	≤0.5	≤0.5	0.5
Levofloxacin	≤0.03 to >4	≤0.03	≤0.03	0.5
Meropenem	≤0.06 to 0.25	≤0.06	≤0.06	0.0
Sparfloxacin	≤0.03 to >1	≤0.03	≤0.03	1.1
Tetracycline	≤0.25 to >4	0.5	1	0.5
Trimethoprim-sulfamethoxazole	≤0.06/1.19 to >2/38	0.12/2.38	>2/38	27.4

a NS, nonsusceptible.

## DISCUSSION

Considered 1 of 12 priority pathogens by the World Health Organization (WHO) (https://www.who.int/medicines/publications/global-priority-list-antibiotic-resistant-bacteria/en/), H. influenzae is a significant cause of invasive disease, causing septicemia, pneumonia, and meningitis. Although the introduction of the Hib conjugate vaccine in 1985, with worldwide adoption over the subsequent decades, has caused a dramatic decline in the number of Hib infections, invasive infections due to other serotypes and nontypeable strains continue to represent a significant burden. In Ontario, Hib became a provincially reportable disease in 1991, with expansion to all types of H. influenzae disease on 1 May 2018 (15, 17). While a previous report documented non-Hib disease in Ontario from 2004 to 2013 (10), the current report summarizes the epidemiology of all cases of invasive H. influenzae in Ontario for the intervening years (2014 to 2018) between the previous report and the introduction of mandatory reporting of all types of invasive H. influenzae in 2018.

While the Hib conjugate vaccine has not completely eliminated Hib (18), the incidence of Hib disease in Ontario remains low, with an overall incidence of 0.04 cases per 100,000 population from 2014 to 2018. This is consistent with the incidence of Hib observed in Ontario during the 9 years preceding this study (0.01 to 0.06 cases per 100,000 population from 2005 to 2013) (https://www.publichealthontario.ca/en/data-and-analysis/infectious-disease/reportable-disease-trends-annually#/21). Similarly low rates of Hib have been reported in the United States (0.03 cases per 100,000 population) (5) and Europe (0.05 cases per 100,000 population) (4, 6, 18). Although the highest age-specific incidence of Hib in Ontario was in infants <1 year old (1.11 cases per 100,000 population), this represented only 8 cases over the 5-year period under investigation. In Ontario, the Hib vaccine is part of a combined vaccine that also includes protection against diphtheria, tetanus, pertussis, and polio (DTaP-IPV-Hib) and is given at 2 months, 4 months, 6 months, and 18 months of age. As part of the deidentification process in this study, age in months was not available to the project team; therefore, it was not possible to determine if the cases of invasive Hib disease occurred among infants before or after completion of the primary vaccine series. The immunization history of children was also not available, although over the period of 2015 to 2018, most infant cases of Hib in Ontario occurred in children who were either unimmunized or had not received the recommended number of Hib-containing vaccines as per their age (https://www.publichealthontario.ca/en/data-and-analysis/infectious-disease/reportable-disease-trends-annually#/21).

Despite a sustained low incidence of Hib, the overall incidence of invasive H. influenzae infections in Ontario increased significantly from 2014 to 2018, from 1.67 to 2.06 cases per 100,000 population, representing a 5.6% APC, largely due to a 7.6% annual increase in H. influenzae in the ≥65-year-old population. In Ontario, this represents the continuation of a trend that saw the incidence of invasive non-Hib infections increase from 0.67 cases in 2004 to 1.60 cases per 100,000 population in 2013 (10), reaching an incidence of 2.00 cases of non-Hib infection per 100,000 population and an overall incidence of 2.06 cases of H. influenzae infection per 100,000 population in 2018. An increasing incidence of invasive H. influenzae disease in the total population and/or in elderly individuals has been documented in several other countries in recent years (4, 5, 7, 9). In Ontario, there was minimal regional variation in the H. influenzae incidence across the province, and the majority of H. influenzae disease was attributed to NTHi strains and, to a much lesser extent, Hia and Hif strains. While NTHi also predominates the invasive H. influenzae landscape in other countries, Hia is rare in Europe, with Hif and Hib comprising the majority of encapsulated strains (4, 6–9, 11).

As with other studies, H. influenzae disease in Ontario disproportionately affects infants aged <1 and those ≥65 years of age. In the very young, invasive H. influenzae disease is caused by a combination of NTHi, Hia, and Hib strains. Other studies have also reported a high incidence of H. influenzae disease and a mixed distribution of serotypes and NTHi strains in the very young (4, 5, 7, 11, 19). In this study, the specimen source of H. influenzae for those aged <1 was primarily blood, but a significant number of strains were isolated from CSF, demonstrating a predilection for the central nervous system in this age group. Concordantly, Whittaker et al. (4) noted that meningitis was much more common in infants aged <1 than in other age groups when the infection was caused by encapsulated strains.

From 2014 to 2018, half of all invasive H. influenzae infections in Ontario occurred in those aged ≥65 years of age, with no observable patterns in the geographic distribution of the incidence across the province. This age group exhibited an elevated incidence of invasive H. influenzae disease compared to younger adults which has also been noted in other countries. Among these individuals, invasive H. influenzae cases are dominated by NTHi strains, followed distantly by Hif strains, and the incidence in this age group has increased over the last 20 years in Ontario and in other regions worldwide (4–7, 9–11). The vast majority of H. influenzae strains in this age group were isolated from blood consistent with European data, which demonstrates that the majority of cases of invasive H. influenzae present as septicemia or pneumonia in those aged ≥60 years of age (4). In the elderly, invasive H. influenzae disease typically occurs in those with underlying health conditions, culminating in a 20% mortality rate (20, 21). The increasing trend of H. influenzae infection in those aged ≥65 years old in Ontario may be due to an aging population, greater use of immunosuppressive treatments, and/or enhanced surveillance and case ascertainment (4). Due to the genetic diversity of NTHi strains, surveillance may benefit from enhanced genetic typing (4, 22), and a vaccine against NTHi, while desirable, may be difficult to produce (23).

Beyond serotype determination, additional genetic typing, such as multilocus sequence typing (MLST), can elucidate the population structure of invasive H. influenzae isolates and reveal possible associations between clinical characteristics and sequence types (7, 24–26). Although we have not generated MLST profiles for the Ontario H. influenzae isolates in this study, exploring further strain relatedness studies is a promising future research direction.

Previous studies, including reports from Ontario, have documented an elevated incidence of invasive H. influenzae disease in those with North American and Australian Indigenous ancestry compared to non-Indigenous people, particularly among children (5, 11, 19, 27, 28). Specifically, several reports have suggested that Hia disproportionately afflicts individuals of Indigenous ancestry, causing serious invasive disease similar to that caused by Hib in young children and infants (29–35). This association could not be explored in this study, as information on patient ancestry was not available.

According to the WHO, H. influenzae is considered a high-priority antimicrobial-resistant pathogen due to concern over the rising resistance rates, threatening treatment options. Treatment of H. influenzae infections depends on the location and severity of the infection. Severe infections such as meningitis and epiglottitis are treated with third-generation cephalosporins such as ceftriaxone or cefotaxime. Other infections may be treated with alternate antibiotics, including amoxicillin/clavulanate, azithromycin, or fluoroquinolones (36). Of particular concern, resistance to ampicillin and other beta-lactams can be acquired through beta-lactamase-containing mobile genetic elements and/or intrinsic resistance via point mutations or recombination, causing upregulation of the AcrAB efflux pumps and/or alteration of the penicillin-binding protein 3. Intrinsic resistance to ampicillin is increasingly common; however, high-level intrinsically resistant strains may also be resistant to cephalosporins and carbapenems (12). Although the rates of resistance of NTHi to ampicillin approach 50% and appear to be increasing in some countries (12), NTHi ampicillin resistance in Ontario remains essentially unchanged, from 26.4% in the 2004 to 2013 study (10) to 27.2% in this study (2014 to 2018). We also noted a significant level of ampicillin resistance in Hib (20.0%) and Hie (16.7%) cases in Ontario. Ampicillin resistance appeared to be mediated at least in part by beta-lactamase activity in the vast majority (99.3%) of ampicillin-resistant isolates. Most Hia and Hif isolates were pansusceptible to the drugs tested, as noted elsewhere (37). However, substantial rates of resistance to clarithromycin, tetracycline, and trimethoprim-sulfamethoxazole were recorded, particularly among Hib, Hie, and NTHi isolates, in Ontario. Together with ampicillin, these drugs should not be used for empirical therapy of severe H. influenzae infections. Resistance to ceftriaxone and meropenem was nonexistent in Ontario, supporting their continued use for treatment of severe infections, including meningitis and sepsis (38). However, reports of carbapenem- or cephalosporin-nonsusceptible H. influenzae strains in other countries (13, 14) necessitate continued surveillance.

As with any observational study, there are limitations inherent to the design. While PHOL conducts the serotyping of >90% of H. influenzae cases in Ontario, isolates not sent for serotyping were not included. Slide agglutination serotyping can produce false results compared to PCR-based capsule typing. However, utilizing standardized reagents and procedures and routine quality control, as employed in this work, dramatically improves the assay performance and accuracy, demonstrating that it can be a valid and reliable serotyping method when performed correctly (39). No data were available on underlying medical conditions, ethnicity, disease presentation, immunization history, prescribed antimicrobial treatment, or patient outcomes; consequently, these data could not be correlated with serotype or antimicrobial resistance. A residential patient postal code was missing in 30% of cases, which may have resulted in an underestimate of the incidence in certain health regions of the province.

Combined with data from Desai et al. (10), we document an increase in invasive H. influenzae disease in Ontario from 2004 to 2018. Individuals <1 year and those ≥65 years of age are the most vulnerable, and incidence among the elderly increased year upon year over the study period. Given that the bulk of H. influenzae disease was caused by NTHi strains, additional genotyping beyond serotyping may be helpful in describing H. influenzae epidemiology. Global trends of antimicrobial resistance in H. influenzae strains require continued vigilance to support appropriate antibiotic stewardship. In May 2018, all invasive H. influenzae, including non-Hib, cases were included in the list of diseases of public health significance (i.e., reportable diseases), which will allow for improved data quality to monitor the incidence and guide appropriate empirical antimicrobial treatment for this segment of the Canadian population.

## MATERIALS AND METHODS

To investigate the epidemiology of invasive H. influenzae disease in Ontario from 2014 to 2018, we described isolates of H. influenzae received by PHOL between 1 January 2014 and 31 December 2018; invasive isolates were defined as those cultured from sterile body sites. PHOL is the provincial reference laboratory and conducts the majority of serotyping (>90%) of H. influenzae cases in Ontario. Serotyping of invasive H. influenzae isolates was necessary in order to meet provincial reporting requirements for diseases of public health significance to identify Hib (2014 to 2018) or all serotypes (after May 2018). In addition to the serotype, the following information was recorded for each isolate: the specimen source, the date the specimen was received, and the patient’s name, age, gender, and residential postal code.

All isolates were identified and confirmed as H. influenzae by matrix-assisted laser desorption ionization–time of flight mass spectrometry (MALDI-TOF MS) using the Bruker MALDI-TOF MS (Bruker Daltonics, Billerica, MA, USA) and the MALDI Biotyper reference library v7 (Bruker Daltonics). Serotyping was performed by the slide agglutination method using antisera to capsular antigens a through f (Thermo Fisher Scientific, Nepean, ON) (40). In addition, antimicrobial susceptibility testing (AST) was performed on 1,261 isolates using the broth microdilution method (Sensititre Haemophilus and Streptococcus HPB1 AST plate; Thermo Fisher Scientific) with MICs interpreted based on the CLSI breakpoints (41, 42). The isolates (*n* = 1,263) were also tested for beta-lactamase activity using nitrocefin solution (prepared from powder; Thermo Fisher Scientific Oxoid, Nepean, ON) according to the manufacturer’s instructions.

The incidence per 100,000 population was calculated using estimates and projections of annual population denominators obtained from Public Health Ontario based on data from Statistics Canada (16). Temporal trends in incidence were calculated using Joinpoint v4.8.0.1 (43); *P* values of ≤0.05 were considered significant. Chi-square tests and Bonferroni-corrected *post hoc* analyses were conducted using R v4.0.3 (44) with the packages chisq.posthoc.test (45) and rcompanion (46). For geographic analysis and mapping, H. influenzae cases were assigned to a public health unit (PHU) based upon the postal code of the patient’s residence for the isolates where the patient’s residential postal code was known. Maps were generated using the custom Public Health Ontario-developed mapping program Easy Maps v2.0.

Ethics and privacy approval for this study was granted by Public Health Ontario’s ethics review board and privacy office, respectively. All study data were deidentified before analysis.
