# Supplementary material for: Increased Incidence of Invasive Haemophilus influenzae Disease Driven by Non-Type B Isolates in Ontario, Canada, 2014 to 2018
Source: Microbiol Spectr. 2021 Oct 6;9(2):e00803-21. doi: 10.1128/Spectrum.00803-21 (PMC8510165; doi:10.1128/Spectrum.00803-21)
Supplement: Supplemental file 1 — Supplemental material. Download SPECTRUM00803-21_Supp_1_seq9.pdf, PDF file, 2.4 MB [file spectrum00803-21_supp_1_seq9.pdf]

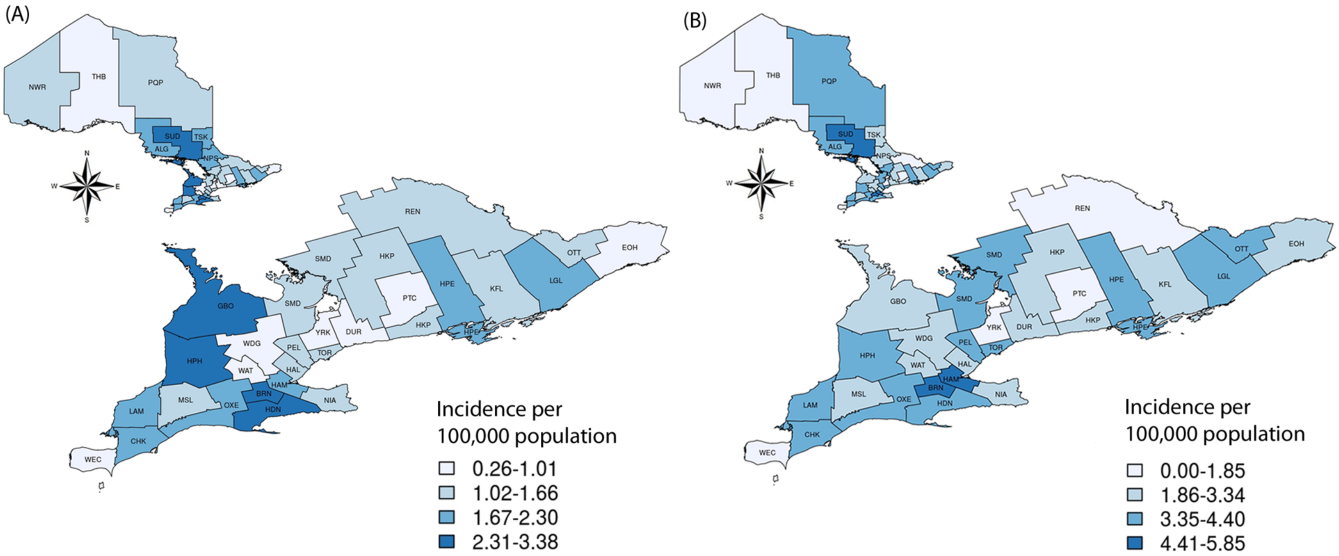

Health Unit

Code	Name	Code	Name
ALG	Algoma District	NPS	North Bay Parry Sound District
BRN	Brant County	NWR	Northwestern
CHK	Chatham-Kent	OTT	City of Ottawa
DUR	Durham Regional	OXE	Oxford Elgin St.Thomas
EOH	Eastern Ontario	PEL	Peel Regional
GBO	Grey Bruce	PQP	Porcupine
HAL	Halton Regional	PTC	Peterborough County-City
HAM	City of Hamilton	REN	Renfrew County and District
HDN	Haldimand-Norfolk	SMD	Simcoe Muskoka District
HKP	Haliburton-Kawartha-Pine Ridge District	SUD	Sudbury and District
HPE	Hastings and Prince Edward Counties	THB	Thunder Bay District
HPH	Huron Perth	TOR	City of Toronto
KFL	Kingston-Frontenac and Lennox and Addington	TSK	Timiskaming
LAM	Lambton	WAT	Waterloo
LGL	Leeds-Grenville and Lanark District	WDG	Wellington-Dufferin-Guelph
MSL	Middlesex-London	WEC	Windsor-Essex County
NIA	Niagara Regional Area	YRK	York Regional

Figure S1: Geographical distribution of incidence of Hi reported in 34 PHUs in Ontario for (A) all cases of Hi (B) cases of NTHi in individuals 65 years of age and older
